# Stereotactic body radiotherapy for adenoid cystic carcinoma metastatic to the lung: a case report

**DOI:** 10.1186/s13256-021-02781-x

**Published:** 2021-04-11

**Authors:** Daijiro Kobayashi, Takanori Abe, Jun-ichi Saitoh, Takahiro Oike, Hiro Sato, Atsushi Musha, Tatsuji Mizukami, Tsuneo Shimizu, Takashi Nakano, Tatsuya Ohno

**Affiliations:** 1 CyberKnife Center, Kanto Neurosurgical Hospital, 1120 Dai, Kumagaya, Saitama 360-0804 Japan; 2 Department of Radiation Oncology, Gunma Prefectural Cancer Center, 617-1 Takahayashi-nishicho, Ota, Gunma 373-8550 Japan; 3 Department of Radiation Oncology, Saitama Medical University International Medical Center, 1397-1 Yamane, Hidaka, Saitama 350-1298 Japan; 4 Division of Radiation Oncology, Department of Radiology, Faculty of Medicine, Academic Assembly, University of Toyama, 2630 Sugitani, Toyama, Toyama 930-0194 Japan; 5 Department of Radiation Oncology, Gunma University Graduate School of Medicine, 3-39-22 Showa-machi, Maebashi, Gunma 371-8511 Japan; 6 Gunma University Heavy Ion Medical Center, 3-39-22 Showa-machi, Maebashi, Gunma 371-8511 Japan; 7 National Institute of Radiological Sciences, National Institute for Quantum and Radiological Science and Technology, 4-9-1 Anagawa, Inage, Chiba 263-8555 Japan

**Keywords:** Adenoid cystic carcinoma, Lung metastases, Stereotactic body radiotherapy, Radiation pneumonitis, Case report

## Abstract

**Background:**

Adenoid cystic carcinoma (ACC) is a rare malignant tumor involving mostly the head and neck region, and frequently the salivary glands. The development of lung metastasis after treatment of the primary tumor is a common occurrence in ACC. Although lung metastases show a slow rate of growth, approximately 10% of patients die from distant metastases. The radioresistance of ACC limits the efficacy of conventional radiotherapy for lung metastases, and the optimal dose remains to be determined. Stereotactic body radiotherapy (SBRT) using CyberKnife can deliver a high dose to the lung tumor, while sparing the surrounding normal lung tissues, leading to favorable local control in non-squamous cell lung cancer and metastatic lung tumors. We report a case of lung metastases from ACC treated successfully with SBRT using CyberKnife.

**Case presentation:**

A 76-year-old Japanese man with ACC who was treated with carbon ion radiotherapy for a primary oropharynx tumor presented with three metastatic lesions in the lung. The tumor masses were located in the right upper, right lower, and left lower lobes of the lungs. Surgical resection was not indicated because of the presence of multiple tumors. The patient underwent SBRT at 60 Gy in 10 sequential fractions for each tumor. The biologically effective dose based on an alpha/beta ratio of 2 Gy was 240 Gy per tumor. The percentage of the total lung volume irradiated with >20 Gy was 4.9%, 3.2%, and 2.6% for each tumor. The patient developed acute radiation pneumonitis during the initial therapy, which resolved at 6 months after the CyberKnife treatment. At 21 months after the first CyberKnife treatment, three tumors showed no signs of recurrence. No late toxicity was observed.

**Conclusions:**

SBRT using CyberKnife is an effective and feasible approach to the management of multiple lung metastases of ACC.

## Background

Adenoid cystic carcinoma (ACC) is a rare malignant tumor arising mostly in the head and neck region that commonly involves the salivary glands [1]. The standard treatment for primary ACC is surgery in most patients [1]. Approximately 40–60% of patients with ACC develop distant metastases at ≥10 years after diagnosis [2, 3]. The most frequent site of distant metastases is the lung [4], and distant metastasis accounts for approximately 10% of the mortality from ACC [1]. Lung metastases progress at a slow rate, and some patients survive for several years if the local disease is controlled effectively. Solitary metastatic tumors can be resected by surgery. Systemic chemotherapy has shown limited benefits in ACC [5].

Although radiotherapy is used in the treatment of lung metastasis, the radioresistant nature of ACC limits its efficacy [6]. Dose escalation increases the probability of local control (LC) but also increases the risk of severe lung toxicity. Stereotactic body radiotherapy (SBRT) is a treatment option for early-stage lung tumors [7]. CyberKnife (Accuray, Sunnyvale, CA, USA) is a type of SBRT that provides excellent dose localization, thereby minimizing the radiation exposure to normal tissues. The efficacy of the method for early-stage lung cancers has been widely reported [7]. Positive results of CyberKnife treatment have been reported in primary and metastatic lung cancers; however, its effect on the clinical course of multiple lung metastases from ACC has not been investigated. Assessing the incidence of acute and late lung toxicities associated with CyberKnife treatment and exploring tumor control aspects are thus important. Here, we present a case of ACC lung metastases successfully treated with CyberKnife.

## Case presentation

**Fig. 1:**
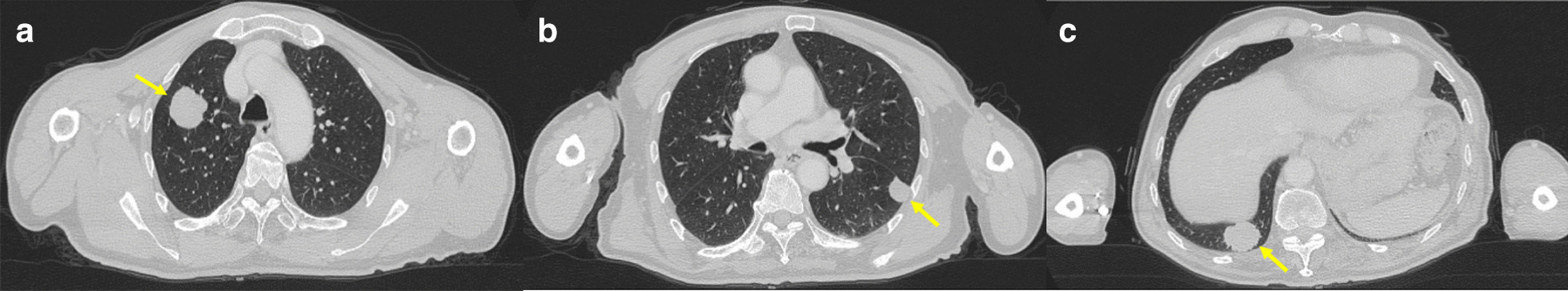
Computed tomography images of metastatic adenoid cystic carcinoma of the lung before CyberKnife: **a** right upper lung, **b** left lower lung, and **c** right lower lung. Arrows indicate tumors

**Fig. 2:**
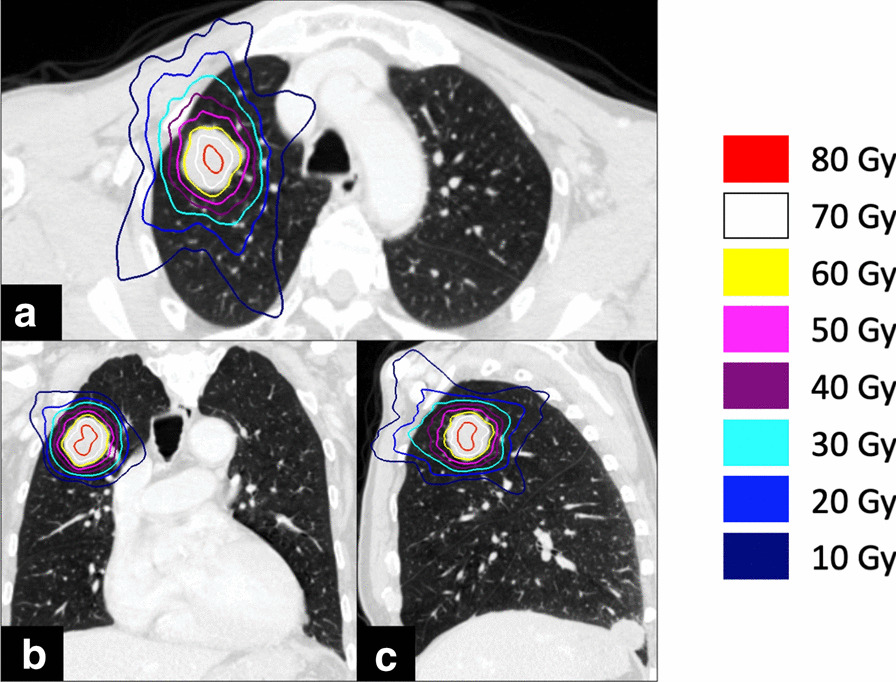
Dose distribution of the CyberKnife treatment for the right upper lung tumor: **a** axial image, **b** coronal image, and **c** sagittal image

A 76-year-old Japanese man with right oropharynx ACC presented at a general hospital. Computed tomography (CT) detected masses of 23 × 19 mm on the right upper lung lobe, 14 × 12 mm on the right lower lung lobe, and 15 × 14 mm on the left lower lung lobe. ^18^F-fluorodeoxyglucose positron emission tomography (FDG-PET) revealed abnormal accumulation in the lung tumors. The patient was diagnosed with T3N0M1, stage IVC ACC of the right oropharynx with lung metastases. Despite the lung metastasis, a favorable long-term prognosis was possible after control of the primary tumor. Radical surgery was not indicated for reasons related to functional preservation, and the patient received carbon ion (C-ion) radiotherapy for the primary tumor. The total dose was 64 Gy (relative biological effectiveness) delivered in 16 fractions. After 30 months, there was no recurrence of the primary lesion; however, the metastatic lung tumors had increased in size. The masses had grown to 32 × 30 mm on the right upper lung lobe, 31 × 25 mm on the right lower lung lobe, and 18 × 17 mm on the left lower lung lobe (Fig. 1). The patient consented to sequential treatment with CyberKnife for multiple lung metastases (Table 1). CT images of 1 mm thickness were acquired to plan the treatment. Respiratory migration was assessed by four-dimensional CT. The gross tumor volume (GTV) was delineated based on thin-slice CT images. The clinical target volume (CTV) was identical to the GTV (CTV = GTV). The planning target volume (PTV) included 2 mm margins surrounding the CTV. The organs at risk (OARs; lung, spinal cord, heart, and skin) were outlined on the treatment planning CT scan and dose–volume histogram analysis. D95 was defined as the minimum dose covering 95% of the GTV for the first and the second CyberKnife treatments or the PTV for the third CyberKnife treatment. The dose prescribed for the PTV was used for the third treatment because of its small size. The patient received a total dose of 60 Gy in 10 fractions of D95. The treatment was planned using the MultiPlan System (Accuray). The composite dose distribution is depicted in Fig. 2. The treatment data for the first CyberKnife administration are summarized in Table 2. Conformity and homogeneity indices were calculated according to the following formulas [8]:

**Table 1:** Treatment data

Treatment number	1st	2nd	3rd*
Tumor location	Right upper lung lobe	Right lower lung lobe	Left lower lung lobe
Tumor volume (cc)	18.5	13.0	12.5
No. of beams	65	71	90
Conformity index	1.09	1.33	1.18
Homogeneity index	1.45	1.54	1.61
Prescribed dose for D95 (Gy)	60	60	60
Prescribed dose for D95 (Gy(EQD2))	80	80	80
Dmax (Gy)	87.0	92.3	96.8
V20 for lung (%)	4.9	3.2	2.6

*In the third treatment, the treatment dose was prescribed for the planning target volume (PTV). PTV had margins of 2 mm added around the gross tumor volume

**Table 2:** Timeline

The event	Timeline
Initial presentation	*T* = 0
Radiological investigations (CT scan and MRI)	*T* = 1 month
Carbon ion radiotherapy for the primary tumor	*T* = 2 months
Increase in the size of metastatic lung tumors	*T* = 32 months
First CyberKnife for right upper lung tumor	*T* = 32 months
Radiation pneumonitis grade 1	*T* = 35 months
Second CyberKnife for right lower lung tumor	*T* = 38 months
Third CyberKnife for left lower lung tumor	*T* = 47 months
No signs of recurrence or no adverse events	*T* = 53 months

*CT* computed tomography, *MRI* magnetic resonance imaging

Conformity index = TV_RI_/TV

Homogeneity index = maximum dose/prescribed dose

TV_RI_ = target volume covered by the reference isodose

TV = target volume

The conformity and homogeneity indices were 1.09 and 1.45, respectively.

**Fig. 3:**
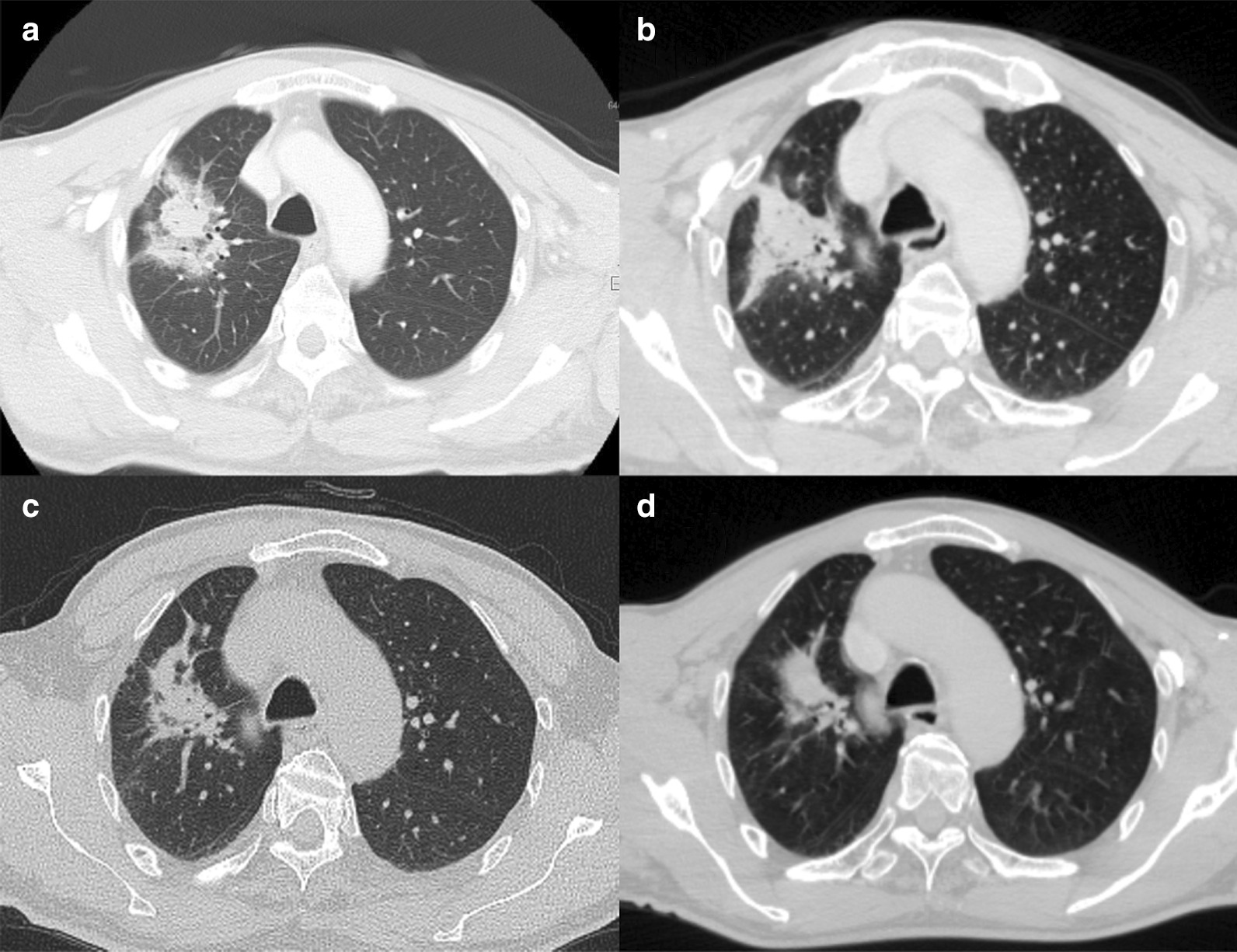
Radiographical course of radiation pneumonitis developed after CyberKnife treatment of the right upper lung tumor. The extent of radiation pneumonitis was limited and it has remained stable for 21 months. **a** 3 months, **b** 6 months, **c** 15 months, and **d** 21 months after CyberKnife

At 3 months after the first CyberKnife treatment, the patient developed acute radiation pneumonitis, which was classified as grade 1 based on the Common Terminology Criteria for Adverse Events, version 4.0 (Fig. 3). The radiation pneumonitis remained at grade 1 for 6 months after CyberKnife treatment. After confirming that the pulmonary fibrosis and inflammatory reaction had stabilized, the second and third tumors in the right and left lower lung lobes were treated at 6 and 15 months after the first CyberKnife treatment, respectively. The patient developed grade 1 pleural effusion after the second CyberKnife session, whereas no adverse events were observed after the third treatment.

There were no chronic adverse events resulting from any of the CyberKnife treatments. Other adverse events, such as esophagitis, rib fractures, pleuritic pain, or bronchial fistula, were not observed. CT or FDG-PET/CT did not detect any signs of recurrence at 21 months after the first CyberKnife session or at 6 months after the third CyberKnife treatment.

## Discussion and conclusions

Patients with ACC may develop distant metastases late in the course of the disease without recurrence of the primary tumor [3]. If the local tumor is controlled, the cause-specific survival decreases in patients with distant metastases [1]. Among patients with head and neck ACC who are treated with C-ion radiotherapy, 15% develop lung metastasis [9]. Girelli *et al*. reported that the extent of resection of metastatic lung tumors contributes significantly to a longer overall survival (OS) in patients with ACC; the 5-year OS rate is 69.5% after complete resection and 51.3% after incomplete resection (*p* = 0.004) [10]. The study underscores the importance of controlling lung metastases to improve the OS of ACC patients. In the present case, although the primary oropharynx ACC was controlled by C-ion radiotherapy, the size of the three metastatic lung tumors increased gradually. Local treatment for multiple lung metastases was sequentially attempted using SBRT based on the efficacy of this therapy for the treatment of lung tumors [7].

The optimal dose regimen for lung metastasis of ACC remains to be determined. Previous studies suggest that 60 Gy (EQD2) is insufficient for the LC of primary pulmonary ACC (Table 3) [11–18]. In addition, the results of conventional radiotherapy for ACC of the head and neck remain inadequate, with a 5-year LC of 56% [19]. Current data indicate that dose escalation is needed to improve LC. However, in conventional radiotherapy, dose escalation also increases the risk of radiation pneumonitis. Other potential adverse events observed after short-term follow-up include grade 1 bronchial stricture and grade 3 esophagitis [11, 18]. Grade 1 bronchial stricture was observed after intensity-modulated radiotherapy with a total dose of 54 Gy delivered in 30 fractions. The biologically effective dose determined by assuming an alpha/beta ratio of 2 Gy (BED2Gy) was 102.6 Gy. Grade 3 esophagitis was observed after concurrent chemoradiotherapy with a total dose of 65 Gy delivered in 35 fractions (BED2Gy = 125.4 Gy) and platinum chemotherapy three times per week. CyberKnife can achieve high-dose irradiation to metastatic ACC of the lung while sparing the surrounding normal lung tissues [20], which improves the control rate. Franzes *et al*. reported good LC following SBRT for oligometastatic salivary gland cancer involving ACC [21]. They used 20–54 Gy in 1–5 fractions (BED2Gy = 94.5–345.6 Gy). In the present study, BED2Gy was 240.0 Gy after administration of 60 Gy in 10 fractions. The dose concentration of CyberKnife is an important consideration. We used prescribed doses to the D95, and the dose to the central PTV was 86.95 Gy (BED2Gy = 465.0 Gy). The high doses of radiation used by CyberKnife allow the delivery of a high BED2Gy to the center of the tumor. In the present case, the high BED2Gy might have contributed to the control of the three tumors at the time of the study.

**Table 3:** Review of the literature on radiotherapy outcomes of pulmonary ACC

Authors	No. of patients	Radiotherapy dose, BED2Gy	Follow-up (months)	Combination therapy	Treatment outcome	Adverse events
Bhandari V [11]	1	54 Gy/30fr., 102.6 Gy	8	–	No evidence of recurrence	Bronchial stricture grade 1
Das S [13]	1	66 Gy/33 fr., 132 Gy	38	–	Local recurrence	NR
Haresh KP [14]	1	60 Gy/30 fr., 120 Gy	19	–	No evidence of recurrence	NR
Kanematsu T [15]	5	50–70 Gy (median 60 Gy), 100–140 Gy (median 120 Gy)	NR	–	Local recurrence in all five cases (5-year OS, 40%; 10-year OS, 0%)	NR
Kim B [16]	1	66 Gy/33 fr., 132 Gy	20	Paclitaxel, cisplatin, docetaxel, gefitinib	Dead without local recurrence	Radiation pneumonitis grade 1
Lee JH [17]	12	60 Gy/30 fr., 120 Gy	8–167 (median 59)	–	5 year OS, 54%; 10-year OS, 27%	NR
Liu J [18]	1	65 Gy/35 fr. + endobronchial boost 10 Gy/1 fr., 125.4 Gy + 60 Gy	90	Cisplatin	Local recurrence	Esophagitis grade 3, skin reaction grade 1, and bronchial stricture grade 1

*fr.* fractions, *BED* biologically equivalent dose, *OS* overall survival, *LC* local control, *NR* not reported

Radiation pneumonitis is a common adverse event associated with radiotherapy for lung cancer. In cases of SBRT for multiple metastatic lesions, severe radiation pneumonitis may lead to treatment-related death, and minimizing its occurrence is thus important. The percentage of total lung volume irradiated with >20 Gy (V20Gy) is correlated with the incidence of radiation pneumonitis [22]. In the present study, the V20Gy(EQD2) values were 4.9%, 3.2%, and 2.6% at the first, second, and third treatments, respectively. These values are sufficient to lower the risk of radiation pneumonitis. Certain factors are associated with increased toxicity, such as centrally located tumors, chemotherapy, and target diameters [23]. If these factors are present, it may be beneficial to increase the number of fractions or decrease the prescribed dose. Pulmonary fibrosis, which is a common response to large doses per fraction in SBRT [24], can make it difficult to assess tumor recurrence and late adverse effects. In the present case, CT and blood tests were performed after the initial treatment. Figure 3 shows the radiographical course of radiation pneumonitis and the limitations in the management of multiple lung metastases after CyberKnife; for instance, LC could not be evaluated due to fibrosis. Thus, FDG-PET/CT imaging would help evaluate the metabolic complete response. Twenty-one months have elapsed since the first treatment, and severe pneumonitis has not occurred. These results suggest that sequential SBRT for multiple lesions can be performed safely with appropriate follow-up periods between treatments.

In conclusion, SBRT using CyberKnife is a feasible treatment for multiple lung metastases of ACC. Although a longer follow-up is necessary to determine the optimal dosing and long-term prognosis, the technique could serve as an effective option to manage lung metastases of ACC.

## Data Availability

The data generated and analyzed in the current study are not publicly available due to the personal patient data included, but are available from the corresponding author on reasonable request.

## Abbreviations

ACC: Adenoid cystic carcinoma
LC: Local control
SBRT: Stereotactic body radiotherapy
CT: Computed tomography
C-ion radiotherapy: Carbon ion radiotherapy
GTV: Gross tumor volume
CTV: Clinical target volume
PTV: Planning target volume
OARs: Organs at risk
OS: Overall survival
BED10: The biologically effective dose, assuming the alpha/beta ratio to be 10
